# Willingness to receive institutional and community-based eldercare among the rural elderly in China

**DOI:** 10.1371/journal.pone.0225314

**Published:** 2019-11-22

**Authors:** Zi-Wei Liu, Yu Yu, Liang Fang, Mi Hu, Liang Zhou, Shui-Yuan Xiao

**Affiliations:** 1 Department of Social Medicine and Health Management, Xiangya School of Public Health, Central South University, Changsha, Hunan, China; 2 Mental Health Institute of the Second Xiangya Hospital, Central South University, National Clinical Research Center on Mental Disorders & National Technology Institute on Mental Disorders, Hunan Key Laboratory of Psychiatry and Mental Health, Changsha, Hunan, China; 3 Hospital Evaluation Office, Xiangya Hospital, Central South University, Changsha, Hunan, China; 4 The Affiliated Brain Hospital of Guangzhou Medical University (Guangzhou Huiai Hospital), Liwan District, Guangzhou, China; Institute of Mental Health, SINGAPORE

## Abstract

Studies on the willingness to receive institutional eldercare among the rural elderly are scarce. This study aims to explore factors associated with the willingness to receive institutional eldercare and community-based eldercare among the rural elderly. A cross-sectional study was conducted in three rural villages of Changde City, Hunan Province, China. A total of 517 elderly were recruited through multistage sampling from these villages. The dependent variable is the willingness to receive eldercare from family (as reference), institution, and community. The independent variables includes sociodemographic characteristics: having physical disease, depression, anxiety, and daily living activities, and concerns toward home-based, institutional, and community-based care, respectively. Results show that 78.3% of the elderly are willing to receive home-based eldercare, 10.8% institutional eldercare, and 8.5% community-based eldercare. The factors associated with the willingness to receive institutional eldercare are having concerns toward home-based (OR = 4.85, P<0.001) and institutional eldercare (OR = 5.51, P<0.001). The factors associated with community-based care is living alone (OR = 2.18, P = 0.034). Finally, the major concerns toward home-based eldercare are lack of care ability and separation of family members, whereas those toward institutional eldercare are unaffordable services and fear of being abandoned by the children. The major concerns toward community-based eldercare includes affordability and lack of necessary services. In summary, elderly having concerns toward home-based care and having no concerns about institutional care are willing to accept institutional eldercare. Elderly who are living alone is tend to accept community-based care. Unaffordable services and loss of contact with family members are the major concerns of institutional eldercare. Aside from the cost, the lack of necessary care services is also a serious concern of community-based eldercare.

## Introduction

The decreasing birth rate and increasing migration of young labor force from rural areas to urban areas have contributed to the huge shortfall in the supply quantity of caregivers, causing the failure to meet the growing needs of the quickly aging population in rural China. In 2015, the rural elderly (aged 60 years and above) had 3.3 children on average, which decreased by one person compared with that in the 2000 year [1]. By contrast, China’s urban areas embraced an inflow of up to 253 million rural people in 2015, most of whom were young work-age adults and over 90% were from rural areas [2]. As a result, the elderly was forced to take care of themselves and left behind in rural areas. For instance, the elderly in empty-nest family (living alone or with a spouse only) accounted for 23.3% of the rural elderly population in 2015 [3]. Furthermore, the percentage of the elderly increased from 10.1% in 2014 to 10.8% in 2016 and was estimated to rise to 23.9%~26.9% in the next three or four decades, which reflects a huge demand for elder care [4].

Home-based eldercare is the primary means of eldercare in urban and rural communities in China. In home-based eldercare, the elderly live in their own family or offspring’s family [5]. Institutional eldercare is another important means of eldercare. In institutional eldercare, the elderly live at an institution and receive services from the institution, and this service is much better developed well in urban areas than in rural areas [6]. In rural areas, only those elderly with no children, no income, and no relatives would live in an institution administrated by the government [7]. Community-based eldercare, as a novelty eldercare form in China, has been developed in a few developed cities, such as Shanghai [8]. Community-based care is lacking in rural areas. However, institutional and community-based eldercare are important means of social care that would remarkably fill the increasing needs of eldercare, against the shrinking capacity of home-based eldercare in the future.

Learning the attitude or willingness of the rural elderly towards institutional and community-based care is vital for informing the development of social eldercare and health promotion programs and related policies that can enhance the elderly’s’ life satisfaction and quality of life [9]. Identifying the influencing factors associated with these attitude or willingness is important for dividing the elderly into different categories. Therefore, evidence-based decision-making can contribute to the more rational allocation of eldercare resources and to satisfying their needs.

Previous studies investigated the factors influencing the willingness to receive eldercare among the elderly in Chinese population [5, 10, 11]. Socioeconomic and demographic factors, including age, sex, sociocultural beliefs, and economic status, are associated with the willingness to receive eldercare [5, 12]. A study conducted in 641 elderly living in Shanghai City showed that loneliness and stigma reduces elderly’s intention to enroll in an eldercare institution, but self-efficacy is positively associated with this intention [10]. The influencing factors differ between urban and rural elderly. A study conducted in the urban and rural areas of three cities (Harbin, Qiqihar and Jiamusi) in north China showed that the factors that influencing the willingness to receive eldercare for urban elderly are age, house property, and social support, whereas those for rural elderly are having children, house property, and living arrangement [5]. Subjective attitude or concerns toward institutional eldercare is an important factor influencing the elderly’s willingness to receive eldercare in institutions. However, past researchers have not reported the effect of concerns toward institutional eldercare on elderly’s willingness.

Huge differences exist between urban and rural areas in terms of social-cultural and living environments, such as, urban and rural elderly differ in their willingness to live in an eldercare institution [13]. Meanwhile, the services of eldercare institutions in rural areas are lagging far behind those in urban areas. Evidence-based policy- making is highly important to improve the institutional care service capacity and quality in rural areas. Hence, information from empirical studies is necessary. The present study aims to explore the factors influencing the willingness of the elderly to receive institutional and community-based eldercare in rural communities in Hunan, China.

## Materials and methods

### Participants and sampling

This study adopted a cross-sectional study design. Ethical approval was granted by the Institutional Review Board of the Xiangya School of Public Health, Central South University. The target population was residents aged 60 years and above who have lived in rural communities of Changde City, Hunan province, China. Eligibility criteria of participants included being 60 years of age and above at the time of interview and a resident in the survey site for at least 6 months. A multistage cluster-sampling method was adopted to identify participants. In the first stage, one district (Dingcheng) was randomly selected from eight districts or counties. In the second stage, one administrative unit (Huayanxi) was randomly selected from 36 towns/Xiang. In the third stage, three administrative villages (Huayanxi, Fengshan, and Xianchishan) were randomly selected from four administrative villages. Finally, all elders (n = 564) within three villages were invited to participate in the study. Those who were not living in the areas during the research period and those with difficulty in communication due to serious physical or mental illness were excluded, resulting in a final sample of 540 residents. Among the 540 participants, one refused to participate in and one had been lost three times. Three were abandoned the study due to unaccessibility of distance. In sum, 517 completed the surveys with a response rate of 95.7%.

The age of the 517 participants ranged from 60 to 94 years with a mean of 69.3(standard deviation, SD: 7.0) years. Male accounted for 49.9% and female were 50.1%. Elderly with an education level of preliminary school and less accounted for 85.7%. Elderly without spouse accounted for 22.4%.

### Data collection

Each participant obtained the oral informed consent before the beginning of interview. To the best of our knowledge, majority of the participants live in the remote rural areas. In this study, 85.7% of the participants have an educational level of preliminary school and less, a number of them cannot read the written consent. Our survey contained no hurtful questions. In addition, no biological sample was collected and no invasive examination mean was employed. Finally, the participants were allowed to decide whether or not to be interviewed. They were allowed to decide to cease and quit the interview whenever they want. We obtained permission from the Institutional Review Board to collect oral consent. The oral consent had been recorded by an audio recorder.

The survey was conducted from January to April in 2015. Interviewers were composed of two postgraduate students from the Department of Social Medicine and Health Management and four undergraduate students with preventive medicine background. All investigators had received consistent training before the investigation. Investigator training included understanding the objectives of the study, scales, the principle and requirements of the interview, skills of asking questions and the use of words. Interviewers conducted face-to-face interviews with each participant in their household after obtaining oral informed consent. Approximately an hour was spent on the total interview and each household was reimbursed with small gifts (equivalent to about USD $5).

### Measurement

#### Dependent variable

The dependent variable in this study is the willingness to receive eldercare. The willingness to receive institutional or community-based care was assessed by the question “Which are you willing to choose?” with the following options: 0 = “home-based care,” 1 = “living in a nursing home,” 2 = “living in a senior care unit of a hospital,” and 3 = “community-based care.” The responders were allowed to mark only one option.

#### Independent variables

**Socio-demographic information:** Demographic information was collected by a purpose-built questionnaire, including birth date, gender, educational attainment, and marital status. Information regarding living arrangement was collected by the question: “In your house, who are you co-residing with?” with the following optional answers: 0 = “living alone” and 1 = “living with others.” Others included spouse, son, daughter, daughter in law, son in law, adult grandson/granddaughter, juvenile grandson/granddaughter, brothers/sisters, and others relatives. For personal income, we asked all participants about the whole personal income in the last year.

**Physical disease:** Information of physical disease was collected from the self-report of participants with a single-item question of “Do you have any physical diseases in the past year?” with of “0 = No, 1 = Yes”.

**Depression:** Depressive symptoms were measured by using the Chinese version of Patient Health Questionnaire 9 (PHQ-9), which showed good reliability in the rural elderly with a Cronbach alpha coefficient of 0.80 [14]. The cut-off scores of 10 was adopted for screening depression.

**Anxiety:** Anxiety symptoms were measured by using the Chinese version of Generalized Anxiety Disorder Scale (GAD-7) [15]. The total score ranged from 0 to 21, with a score of 10 as the cut-off point for screening positive for anxiety [16].

**Daily living activities:** A simplified version of daily living activities (ADL), which includes a physical self-maintenance scale and an instrumental ADL scale, was adopted to measure the ability of the elderly to perform daily living activities, [17]. The physical self-maintenance scale consists of six items, and the instrumental ADL scale consists of eight items. Each item is scored as follows: I CAN handle it in most of the time = 1 and I CANNOT handle it in most of the time = 0. Total score, which ranges from 0 to14, is the sum of all item scores. A total score of lower than 12 is defined as disabled. In this sample, this scale showed good reliability with a Cronbach alpha of 0.847 and a two-week test-retest stability of 0.731 (P<0.001).

#### Concerns toward eldercare in future

For concerns toward home-based care, we asked each participant “For home-based eldercare, which are your willing to choose?”, with the following options: 0 = “no concern,” 1 = “no one to provide necessary services,” 2 = “lack of accompanying,” 3 = “lack of necessary care equipment,” 4 = “not safe,” and 5 = “increasing the family burden of young adults.” For institutional care, we asked each participant “For eldercare institutions, which are your willing to choose?” with the following options: 0 = “no concern,” 1 = “cost,” 2 = “lack of necessary care service,” 3 = “without visits of children,” 4 = “lack of social activities,” 5 = “stigmatized,” 6 = “loss of care from children,” 7 = “not worth,” 8 = “security problem,” and 9 = “others.” As for community-based eldercare, we asked each participant “For community-based care, which are you willing to choose?” with the following options: 0 = “no concern,” 1 = “cost,” 2 = “lack of necessary care service,” 3 = “without visits of children,” 4 = “lack of social activities,” 5 = “stigmatized,” 6 = “loss of care from children,” 7 = “not worth,” 8 = “security problem,” and 9 = “others.” The responders were allowed to mark as many as they want.

#### Statistical analysis

The proportion and counts of subjects in each group were calculated. Chi-square test was used to examine associations between willingness to receive different kinds of eldercare and demographic variables, physical disease, depression, anxiety and concerns toward eldercare. Factors associated with institutional care or community-based care were identified by using multiple logistic regression (forward: LR), which included variables that were statistically significant at the nominal two-side P < 0. 05 level in the above univariate analyses. Odds ratio (OR) and 95% confident interval (CI) were used to quantify associations SPSS 14.0 software (SPSS/IBM, Chicago, IL) was used in data analysis.

## Results

### General information

**Fig 1:**
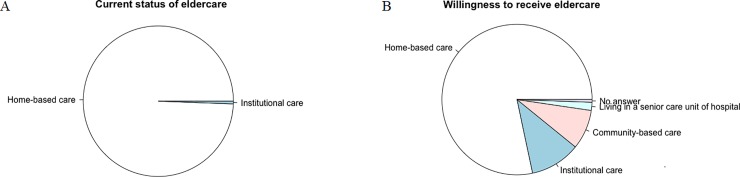
Current status of eldercare and willingness to receive eldercare among elderly. A indicates the current status of eldercare among rural elderly. B indicates the willingness to receive eldercare of rural elderly.

Of the 517 elderly, 99.4% of subjects (n = 514) reported receiving homed-based care at the time of this investigation. Three subjects (0.6%) live in eldercare institutions. No subject reported receiving community-based eldercare (Fig 1A). As for the willingness to receive eldercare, 405 (78.3%) chose home-based care, 56 (10.8) chose institutional eldercare, and 44 (8.5%) chose community-based care. Three participants did not answer this question. Nine participants chose living in a senior care unit of a hospital (Fig 1B). In the following analyses, we only compared the differences in independent variables among the elderly who chose the first three types of eldercare. Those elderly were categorized into three groups: elderly willing to receive home-based eldercare (home-based care group) as a reference group, elderly willing to receive institutional eldercare (institutional care group), and elderly willing to receive community-based care (community-based care).

### Socio-demographic characteristics

The participants differ in living arrangement. The proportions of living alone is higher in the institutional (23.2%) and community care (27.3%) groups than in the home-based care group (14.6%). However, no differences in age, gender, education, marriage, and income are found among the three groups. The detailed socio-demographic information is shown in Table 1.

**Table 1:** Demographic characteristic of participants.

	Total	Willingness to receive home-based care	Willingness to receive institutional care	Willingness to receive community-based care	P
**N**	517	405	56	44	
**Age**					0.158
60–69	57.8 (299)	59.5 (241)	44.6 (25)	56.8 (25)	
70–79	32.9 (170)	30.6 (124)	48.2 (27)	34.1 (15)	
80–94	9.3 (48)	9.9 (40)	7.1 (4)	9.1 (4)	
**Gender**					
Male	258 (49.9)	48.4 (196)	51.8 (29)	61.4 (27)	0.252
Female	259 (50.1)	51.6 (209)	48.2 (27)	38.6 (17)	
**Education**					
Preliminary and less	85.7 (443)	87.4 (354)	80.4 (45)	81.8 (36)	0.118
Junior middle school	11.8 (61)	11.4 (46)	12.5 (7)	13.6 (6)	
Senior middle school & more	2.5 (13)	1.2 (5)	7.1 (4)	4.5 (2)	
**Marriage**					
Married, living with spouse	73.3 (379)	74.8 (303)	67.9 (38)	68.9 (29)	
Married, separated with spouse	4.3 (22)	4.2 (17)	3.6 (2)	6.8 (3)	
Widower	20.9 (108)	19.5 (79)	26.8 (15)	27.3 (12)	
Divorced	1.0 (5)	0.7 (3)	1.8 (1)	0.0 (0)	
Never married	0.5 (3)	0.7 (3)	0.0 (0)	0.0 (0)	
**Living arrangement**					
Living alone	84 (16.2)	14.6 (59)	23.2 (13)	27.3 (12)	0.037
Living with others	421 (81.4)	85.4 (346)	76.8 (43)	72.7 (32)	
**Income (last year, CNY)**					
0–2300	29.4 (152)	30.0 (121)	32.7 (18)	22.7 (10)	0.101
2301–5000	25.1 (130)	27.7 (112)	18.2 (10)	11.4 (5)	
5001–10000	23.2 (120)	21.8 (88)	25.5 (14)	36.4 (16)	
10001–30000	18.4 (95)	17.1 (69)	18.2 (10)	27.3 (12)	
30001-	3.5 (18)	3.5 (14)	5.5 (3)	2.3 (1)	
Missing	0.4 (2)	0	0	0	

CNY, Chinese Yuan.

### Depression, anxiety, physical disease, and ADL

The institutional and community care groups have higher proportions of depression and anxiety than the home-based care group. The proportion of depression is higher in the institutional and community care groups (42.9% and 37.2%, respectively) than in the home-based care group (26.4%). The proportion of anxiety is similar among three groups (P = 0.024). However, ADL (P = 0.166) and having physical disease (P = 0.540) show no difference among the three groups. (Table 2)

**Table 2:** Depression, anxiety and physical disease among the elderly.

	Home-based care	Institutional care	Community-based care	P
**PHQ-9**				
0–9	73.6 (293)	57.1 (32)	62.8 (27)	0.019
10–27	26.4 (105)	42.9 (24)	37.2 (16)	
**GAD-7**				
0–9	89.5 (357)	76.8 (43)	86.0 (37)	0.024
10–27	10.5 (42)	23.2 (13)	14.0 (6)	
**Physical disease**				
Yes	88.6 (359)	94.6 (53)	95.5 (42)	0.166
No	11.4 (46)	5.4 (3)	4.5 (2)	
**ADL**				
Yes	87.7 (355)	89.3 (50)	93.2 (41)	0.540
No	12.3 (50)	10.7 (6)	6.8 (3)	

ADL, daily living activities.

### Concerns toward the three types of eldercare among the rural elderly

The institutional care group has a higher proportion of having concerns about home-based than the home-based and community care groups (P<0.001, see Table 3). However, the former group has a lower proportion of having concerns about institutional care than the other two latter groups. A marginal significance is found in terms of having concerns about community care among the three groups (P = 0.053).

**Table 3:** Concerns toward three types of eldercare among the elderly.

	Home-based care	Institutional care	Community-based care	P
**Having concerns towards home-based care**				
Yes	26.3 (106)	64.3 (36)	38.6 (17)	<0.001
No	73.7 (297)	35.7 (20)	61.4 (27)	
**Having concerns towards institutional care**				
Yes	85.1 (344)	57.1 (32)	77.3 (34)	<0.001
No	14.9 (60)	42.9 (24)	22.7 (10)	
**Having concerns towards community care**				
Yes	38.0 (152)	21.4 (12)	36.4 (16)	0.053
No	62.0 (248)	78.6 (44)	63.6 (28)	

### Factors associated with institutional care and community care among rural elderly

In univariate logistic regression analysis, factors associated with institutional care are depression, anxiety, having concerns towards home-based care, no having concerns towards institutional care and community-based care. However, factor associated with community-based care only includes living alone. (see Table 4)

**Table 4:** The results of univariate logistic regression.

		Institutional care vs home-based care		Community-based care vs home-based care	
		OR (95%CI)	P	OR (95%CI)	P
Living alone	No	1		1	
	Yes	1.77(0.90, 3.49)	0.098	2.20(1.07, 4.51)	0.032
Depression	No	1		1	
	Yes	2.09(1.18, 3.72)	0.012	1.65(0.86, 3.19)	0.134
Anxiety	No	1		1	
	Yes	2.57(1.28, 5.16)	0.008	1.38(0.55, 3.46)	0.494
Having concerns towards home-based care	No	1		1	
	Yes	5.04(2.80, 9.10)	<0.001	0.57(0.30, 1.08)	0.085
Having concerns towards institutional care	Yes	1		1	
	No	4.30(2.37, 7.80)	<0.001	1.69(0.79, 3.59)	0.176
Having concerns towards community care	Yes	1		1	
	No	2.25(1.15, 4.39)	0.018	1.07(0.56, 2.05)	0.832

In multivariable logistic regression analysis, factors significantly associated with institutional care are having concerns toward home-based care (OR = 4.85, P<0.001) and having concerns toward institutional care (OR = 5.51, P<0.001). The former indicates that those elderly having concerns toward home-based care are likely to choose institutional care over home-based care. The latter indicates that the elderly having no concerns toward institutional care are likely to choose institutional care. (Table 5)

**Table 5:** Factors associated with willingness to receive institutional eldercare.

Factors		OR (95%CI)	P
**Variables in the equation**			
Having concerns towards home-based care	No	1	
	Yes	4.85 (2.54, 9.24)	<0.001
Having concerns towards institutional care	Yes	1	
	No	5.51 (2.97 10.25)	<0.001
**Variables not in the equation**			
Living alone			0.611
Having concerns towards community care			0.138
Depression			0.081
Anxiety			0.244

The factor significantly associated with community-based care is living alone (OR = 2.18, P = 0.034), which indicates that the elderly who are living alone are likely to choose community-based care over home-based care. (Table 6)

**Table 6:** Factors associated with willingness to receive community-based eldercare.

Factors		OR (95%CI)	P
**Variable in the equation**			
Living alone	no	1	
	yes	2.18 (1.06, 4.48)	0.034
**Variables not in the equation**			
Having concerns towards home-based care			0.187
Having concerns towards community care			0.819
Having concerns towards institutional care			0.171
Depression			0.159
Anxiety			0.526

### Ranking list of eldercare concerns among the rural elderly

Of the 517 subjects, our study found the major concerns toward home-based care are lack of care ability and separation of family members. The reported top three concerns are “No one to provide care services,” “Lack of accompanying,” and “Lack of necessary care equipment” (See Table 7). As for institutional care, the major concerns are unaffordable services and fear of being abandoned by the children. (Table 8). For community-based eldercare, the major concerns include affordability, lack of necessary care, and not worth to have.

**Table 7:** Detailed concerns toward home-based care among the elderly(n = 346).

	n	Reported rate (%)
No one to provide care services	88	25.4
Lack of accompanying	65	18.7
Lack of necessary care equipment	36	10.4
Not safe	23	6.6
Lack of daily life activities assistance	15	4.3

**Table 8:** Detailed concerns toward institutional and community-based care among the elderly (n = 517).

	Institutional care	Community-based care
Affordability	213(50.7)	85(46.2)
Lack of necessary care services	83(19.8)	26(14.1)
Loss of contacting with offspring	19(4.5)	1(0.5)
Lack of social activities	19(4.5)	5(2.7)
Stigma	87(20.7)	13(7.1)
Loss of offspring’s care	53(12.6)	14(7.6)
Not worthy to go	51(12.1)	17(9.2)
Safety	5(1.2)	1(0.5)

## Discussion

### Main findings

The main findings of the present study are that 78.3% of the elderly are willing to receive home-based eldercare, 10.8% institutional eldercare, and 8.5% community-based eldercare. Multiple logistic regression analysis shows that willingness to receive institutional eldercare is associated with having concerns toward home-based and institutional eldercare. In specific, elderly having concerns about home-based care are tend to choose institutional eldercare (versus home-based care). In addition, elderly having no concerns about institutional care are likely to choose institutional eldercare. Factors associated with community-based care are living alone, which indicates that elderly who are living alone are likely to choose community-based care against home-based care. Finally, the major concerns toward home-based eldercare are lack of care ability and separation of family members. The major concerns toward institutional eldercare are unaffordable services and fear of being abandoned by the children. The major concerns toward community-based eldercare includes affordability, lack of necessary care, and not worth to have.

### Home-based eldercare for rural elderly

In the present study, 78.3% of the rural elderly preferred home-based eldercare. This proportion is significantly higher than the 59.0% of Xing’s study conducted in east north of China [5]. However, both findings indicate that receiving eldercare from family members is still the first choice of the elderly in rural areas, which is in line with the social cultural customs of rural communities. The custom of Chinese social culture stipulates that children must care for their parents. With thousands of years of agricultural civilization, the impact of Confucianism and filial piety was deeply rooted in every Chinese value system with emphasis and advocate on familial loyalty and collectivism. The elderly takes for granted that their offspring have the responsibility and obligation to care for their parents when their parents get old. Another possible explanation is that the elderly needs financial support from their offspring. The rural elderly have no income after not doing farm work. Meanwhile, the subsidy of new rural social endowment insurance for elderly is 65 CNY (around 10 USD) per month from the government, which is insufficient to cover even the basic daily expenditure [18]. Therefore, the elderly live on the money given by their children.

### Institutional eldercare for rural elderly

Our study found that 10.8% of the elderly are willing to receive institutional eldercare, for instance, living in a nursing home. Factors associated with willingness to receive institutional eldercare are having concerns towards home-based care and institutional eldercare. Those elderly having concerns about home-based care are tend to prefer institutional eldercare. In addition, those elderly having no concerns about institutional care are likely to receive institutional eldercare. These associations are reasonable. Furthermore, an important obstacle of willing to receive institutional eldercare is the ability to pay for the services, which has not been reflected in the logistic regression. However, reported concerns toward institutional eldercare can give us some clues, which are discussed below.

### Community-based eldercare for rural elderly

Our study found 8.5% of the elderly are willing to receive community-based eldercare. Community-based eldercare as a novelty eldercare pattern is only planned and offered a few large cities, such as Shanghai. To the best of our knowledge, the conception of community-based eldercare has not been accepted and understood widely in rural areas. The factors associated with community-based care analysis indicates that those elderly who are living alone are likely to choose community-based care against home-based care. Those elderly who have no children, no income, and no relative would be sent to an government-administrating eldercare institution. The budget of government covers the expenditure of those elderly. Therefore, those healthy elderly living in their own home alone are likely to accept community-based eldercare. However, in our investigated villages, no eldercare service is provided by any community. As far as we know, only one Xiang health station and four village clinics (all are the primary medical institutions) in Huayanxi village to provide medical services for 4294 residents, including children, adults, and elderly. In addition, doctors /nurses in rural clinics usually received medical training for 3 years or less [19]. They can only provide several simple medical examination and treatment, such as health checkup, giving conventional drugs or an injection, and basic first aid. Hence, community-based eldercare in rural area still needs improvement.

### Eldercare concerns of rural elderly

Our study found the major concerns toward home-based care were lack of care ability and separation of family members. The major concerns for institutional care were the unaffordable price of care services and fear of being abandoned by the children. We reckoned concerns toward home-based care and institutional care may result from the same reasons: insufficiency of income, loss of social ties from family, and lack of social care. Firstly, the elderly in rural areas have a lower level of subsidy than those elderly living in urban areas. For those elderly living in urban areas, their insurances have three parts: government, enterprises, and social pension. For instance, elderly retiring from governmental agencies have a pension paid by the government. Those retiring from enterprises have basic endowment insurance for the urban working group. For those urban elderly who are not previously employed, social endowment insurance for non-working residents supports their lives. However, elderly pension in rural areas has only one type, namely, the subsidy of new rural social endowment insurance. As a result, the income of the elderly cannot afford the cheapest nursing home. In our investigated villages, no nursing home has been built up and put into operation as far as we know. In the urban area of Changde City where our investigated village is located, the nursing home charges from 6,000 to 22,800 CNY per year, and even the cheapest one is not affordable for over two-third of our participants [20]. Second, elderly are worried about the loss of contacting with family members, such as children. In order to make more money, children have to migrate to cities to find job in a big company. The elderly were forced to separate from their children and left behind in rural areas. Those elderly living in eldercare institutions had similar concerns. For instance, their children may not come to visit them or care about them anymore because they were taken care by the eldercare institutions. Last, the elderly have received almost nothing for social care except for a limited financial subsidy from the government in rural areas. Therefore, various types of social support for the elderly is necessary. In addition, the elderly received limited community-based care. Communities provide no eldercare services for most of elderly, but just minimal financial support for those elderly who have no children [21].

The present study has some limitations to declare. Firstly, the rural elderly were recruited from only one city. Therefore, our finding can only be generalized to the rural areas in central China. Second, qualitative data on barriers associated with willingness to receive institutional eldercare, in particular detailed barriers from inside and outside of family barriers, could facilitate the eldercare service utility in rural areas. However, we did not collect these data. Our subsequent research will use focus group discussion to address this issue. Third, this is a cross-sectional study. Thus, the identified factors are not risk factors or causes. Longitudinal studies are warranted to confirm the casual relationship.

## Conclusions

In this study, 78.3% of the elderly are willing to receive home-based eldercare, 10.8% institutional eldercare, 8.5% community-based eldercare. The factors associated with willingness to receive institutional eldercare are having concern towards home-based and institutional eldercare, respectively. The factors associated with community-based care is living alone. Finally, the major concerns toward home-based eldercare are lack of care ability and loss of contact with family members. The major concerns toward institutional eldercare are the unaffordable services and fear of being abandoned by the children. The major concerns toward community-based eldercare includes unaffordability and lack of necessary care.

## Supporting information

S1 Data (SAV)
